# The effect of salient stimuli on neural oscillations, isometric force, and their coupling

**DOI:** 10.1016/j.neuroimage.2019.05.032

**Published:** 2019-09

**Authors:** Giacomo Novembre, Vijay M. Pawar, Marina Kilintari, Rory J. Bufacchi, Yifei Guo, John C. Rothwell, Gian Domenico Iannetti

**Affiliations:** a Department of Neuroscience, Physiology and Pharmacology, University College London (UCL), UK; b Neuroscience and Behaviour Laboratory, Istituto Italiano di Tecnologia (IIT), Rome, Italy; c Department of Computer Science, University College London (UCL), UK; d Institute of Neurology, University College London (UCL), UK

**Keywords:** Force, EEG, Auditory, Somatosensory, Beta oscillations, Cortico-muscular resonance (CMR)

## Abstract

Survival in a suddenly-changing environment requires animals not only to detect salient stimuli, but also to promptly respond to them by initiating or revising ongoing motor processes. We recently discovered that the large vertex brain potentials elicited by sudden supramodal stimuli are strongly coupled with a multiphasic modulation of isometric force, a phenomenon that we named cortico-muscular resonance (CMR). Here, we extend our investigation of the CMR to the time-frequency domain. We show that (i) both somatosensory and auditory stimuli evoke a number of phase-locked and non-phase-locked modulations of EEG spectral power. Remarkably, (ii) some of these phase-locked and non-phase-locked modulations are also present in the Force spectral power. Finally, (iii) EEG and Force time-frequency responses are correlated in two distinct regions of the power spectrum. An early, low-frequency region (∼4 Hz) reflects the previously-described coupling between the phase-locked EEG vertex potential and force modulations. A late, higher-frequency region (beta-band, ∼20 Hz) reflects a second coupling between the non-phase-locked increase of power observed in both EEG and Force. In both time-frequency regions, coupling was maximal over the sensorimotor cortex contralateral to the hand exerting the force, suggesting an effect of the stimuli on the tonic corticospinal drive. Thus, stimulus-induced CMR occurs across at least two different types of cortical activities, whose functional significance in relation to the motor system should be investigated further. We propose that these different types of corticomuscular coupling are important to alter motor behaviour in response to salient environmental events.

## Highlights

• We delivered salient stimuli to humans exerting a constant isometric force.
• The stimuli evoked several modulations of both EEG and Force spectral power.
• Some of these modulations were coupled across EEG and Force.
• The coupled modulations were both phase-locked (∼4 Hz) and non-phase-locked (∼20 Hz).
• Stimulus-induced corticomuscular coupling involves two types of cortical activity.

## 1 Introduction

The human brain has evolved to cope with changing environments. To do so, appropriate behaviours must be deployed in response to salient sensory events. Thus, sensory and motor systems must cooperate, intertwining the detection of behaviourally-relevant information with the execution of appropriate motor responses (Prinz, 1997; Hommel et al., 2001; Ullsperger et al., 2014; Wessel and Aron, 2017). However, sensory and motor systems are often studied in isolation, and the neurophysiological consequences of sudden environmental changes are mostly interpreted from a sensory perspective (Arnal and Giraud, 2012; Jensen et al., 2012), ignoring the principle that perception ultimately serves and guides action (Wolpert and Kawato, 1998; Horvitz, 2002).

To fill this gap, we recently explored the effect of unexpected sensory events on both electrocortical brain activity and motor output (Novembre et al., 2018). We made two basic observations. First, salient stimuli, regardless of their sensory modality, evoke an involuntary, multiphasic modulation of isometric force exertion. This modulation consists of an initial force decrease (∼100 ms post stimulus), followed by a force increase (∼250 ms), and by another longer lasting force increase (∼350 ms up to ∼2000 ms). Second, we observed that this force modulation is tightly coupled with the well-known EEG vertex potential (or vertex wave) evoked by salient sensory stimuli (Davis, 1939; Bancaud et al., 1953; Mouraux and Iannetti, 2009). We used the term Cortico-Muscular Resonance (CMR) to refer to this basic physiological mechanism, which might subserve the preparation of appropriate behaviour in response to salient stimuli (Novembre et al., 2018).

**Fig. 1:**
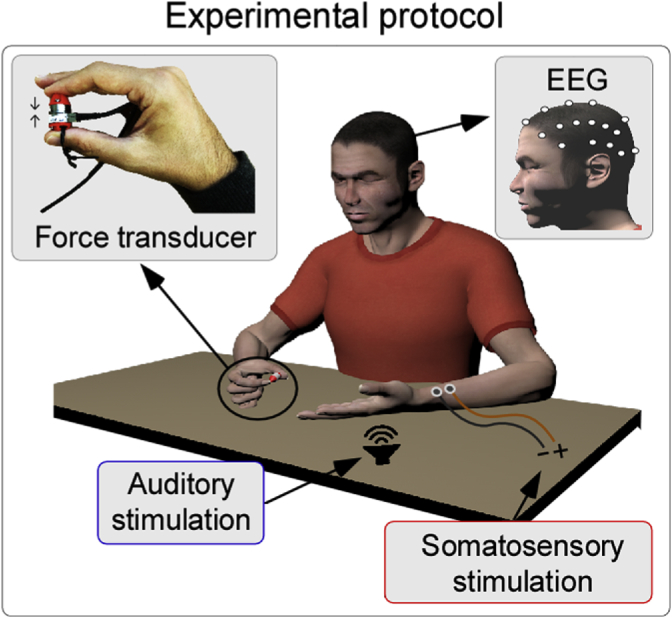
Experimental protocol (adapted from Fig. 1 of Novembre et al., 2018). Participants were instructed to perform an isometric motor task: applying a constant force on a transducer using the thumb and index finger of the right hand, while keeping their eyes closed. Meanwhile, we delivered either somatosensory stimuli (electrical stimulation of the left median nerve) or acoustic stimuli (through a loudspeaker placed close to the participant's left hand). All stimuli were isolated, fast-rising and non-task-relevant. The timing of the stimuli and their order were randomized. EEG and force were recorded simultaneously.

All our previous observations were made in the time domain, and therefore might not capture the full coupling between cortical and muscular processes elicited by salient stimuli. For this reason, here we explored both the brain and the force responses in the time-frequency domain. We simultaneously recorded EEG and Force while human participants were exerting a constant isometric force on a transducer using the thumb and the index finger of the right hand, and receiving isolated, fast-rising, and non-task-relevant somatosensory or auditory stimuli (Fig. 1).

Specifically, we first extensively characterised the modulations of EEG spectral power (i.e., event-related suppression or enhancement of power) induced by somatosensory and auditory stimuli, and distinguished phase-locked from non-phase-locked spectral modulations using the Phase Locking Value (PLV; Lachaux et al., 1999). Second, we used the same analytical approach to explore the modulations of Force spectral power induced by the same stimuli. Finally, given that we observed remarkable similarities between the modulations of EEG and Force power, we explored their coupling by correlating the two spectral powers across trials (“over trials” correlations; Cohen, 2014). This analysis tested whether trials with large modulations of EEG oscillatory power also entailed large modulations of Force oscillatory power at the same latency and frequency. All analyses were conducted using a fully data-driven approach, i.e. forming significant clusters over time points, frequency bins and neighbouring electrodes, corrected for multiple comparisons using a non-parametric cluster-based permutation method (Maris and Oostenveld, 2007).

## 2 Materials and methods

### 2.1 Participants

Twenty-eight right-handed healthy human participants (14 males, mean age [±SD] 23.8 ± 3.3 yrs, age range 19–31 yrs) took part in the experiment. All participants gave written informed consent and were paid for their participation. All procedures were approved by the ethics committee of University College London. A subset of the current dataset was previously analysed in the time-domain (Novembre et al., 2018). All time-frequency analyses presented here are novel and have never been reported before.

### 2.2 Experimental design

Experiments were conducted in a dim, silent, temperature-controlled room. Participants sat in front of a table, with the ulnar aspect of the forearm resting on the table surface. They were asked to exert a constant isometric force on a transducer, which was held between the index finger and thumb of the right hand. While exerting the force, participants received either auditory or somatosensory stimuli. In a short preliminary session, participants were familiarised with the stimuli. Participants were explicitly told that the stimuli were not task related. The experimental setup is illustrated in Fig. 1.

The experiment consisted of 14 blocks. Before each block, participants were instructed to keep their eyes closed (to minimize distraction and reduce eye movements) and exert a gradually increasing force, until they reached a level comprised between 1 and 2 N. Feedback to the participants was provided verbally by the experimenters, who could read the measured force in real time. Once the correct level of force was reached, participants were instructed to keep the applied force as constant as possible, and the block started.

During each block, participants received 5 to 7 auditory or somatosensory stimuli at each of three intensities (low, middle, high), as detailed below. The order of stimulus modality and intensity was randomized, and the inter-stimulus interval was 6–10 s (rectangular distribution). Therefore, the average duration of the applied force in one block was approximately 48 s. There was a short pause of approximately 5–10 s between consecutive blocks. During the pauses, participants had to open their eyes, and to interrupt the force exertion. Each participant received 14 stimuli for each intensity and modality, for a total of 84 stimuli.

### 2.3 Sensory stimuli

Auditory stimuli consisted of a fast-rising tone (rise and fall time 5 ms, frequency 4,000 Hz, duration 50 ms), which was presented through a single CAT LEB-401 loudspeaker placed in front of the left hand of the participant. Somatosensory stimuli were constant-current square-wave electrical pulses (duration 200 μs; Digitimer DS7A) delivered through a pair of cutaneous electrodes placed over the left median nerve at the wrist.

Stimuli were delivered at three intensities: low, middle and high. Stimulus intensity was adjusted individually prior to the beginning of each experiment. High intensity corresponded to the maximal loudness (for auditory stimulation) or current (for electrical stimulations) that each participant could tolerate without feeling discomfort or pain. This was determined by presenting participants with gradually increasing stimulus intensities, starting with bearably noticeable stimuli. Participants were asked whether each stimulus was causing pain or discomfort. The resulting intensity of the high auditory stimuli never exceeded 70 dB of sound pressure level, while the intensity of the high electrical stimulations never exceeded 60 mA (average high electrical stimulation = 26.4 mA). Middle and low stimulus intensities were 60% and 20% of the high stimulus intensity, respectively. All stimuli did not elicit an overt startle response, as further discussed in our previous work describing the current procedure (Novembre et al., 2018).

Stimulus presentation was controlled using the software Presentation (Neurobehavioral systems). Triggers synchronized with stimulus onset were sent to two computers used for acquiring Force and EEG data.

### 2.4 Force recording

The force applied by the participants (see Experimental Design, above) was sampled using a force-torque (F-T) transducer (ATI nano17, Industrial Automation, calibration = SI-12-0.12, resolution = 3.125 mN). This device measures mechanical responses using silicon strain gauges within a monolithic design to provide high stiffness characteristics whilst protecting against noise. The device allows recording six components of force and torque (Fx, Fy, Fz, Tx, Ty, Tz). The ‘Fz’ component represented the direction towards which participants were instructed to exert the force while holding the transducer (Fig. 1), and it was the source of the data reported hereafter. The transducer was connected to a data acquisition card (National Instruments 6363) through which the sensor data from the silicon strain gauges was converted into F-T information based upon calibrated values established by the manufacturer. At the start of each recording session, the F-T information was set to zero to mitigate the effects of potential sensor drifts. Data were sampled at 500 Hz with unique timestamps to allow synchronisation with the stimulation triggers. To facilitate two-finger grip, the transducer was mounted in between two plastic cylindrical extensions (Fig. 1).

### 2.5 EEG recording

The EEG was recorded using a 32-channel amplifier (SD-LTM-32 Express, Micromed Italy) at a sampling rate of 1,024 Hz (hardwired high-pass filter = 0.15 Hz), from 26 Ag–AgCl electrodes placed on the scalp according to the International 10–20 system and referenced to the nose. Electrode positions were ‘Fp1', ‘Fpz’, ‘Fp2', ‘F7', ‘F3', ‘Fz’, ‘F4', ‘F8', ‘T3', ‘C3', ‘Cz’, ‘C4', ‘T4', ‘T5', ‘P3', ‘Pz’, ‘P4', ‘T6', ‘O1', ‘Oz’, ‘O2', ‘FCz’, ‘FC4', ‘FC3', ‘Cp3', ‘Cp4' (Sharbrough et al., 1991). The electro-oculogram (EOG) was recorded from two pairs of surface Ag–AgCl electrodes, with one electrode placed laterally to the outer canthus and the other below the lower eyelid. Impedances were kept below 10 kΩ.

### 2.6 Force data processing

Force magnitude time series were first interpolated to obtain a regular sampling rate of 1,000 ​Hz. Continuous data were band-pass filtered at 0.5–45 ​Hz (Butterworth, third order) and then segmented into epochs of 4 ​s (−1 to +3 ​s relative to stimulus onset). Trials contaminated by artefacts (±0.3 ​N from the mean of the pre-stimulus interval) or deviating more than 4 SDs from the participant's mean exerted force across all trials were excluded from further analyses (this was done separately within each sensory modality and energy). The corresponding EEG trials were also excluded. These trials constituted 18% of the total number of trials.

### 2.7 EEG processing

Continuous EEG data were first band-pass filtered at 0.5–95 ​Hz (Butterworth, third order), and then segmented into epochs of 4 ​s (−1 to +3 ​s relative to stimulus onset). A notch filter (47–53 ​Hz, Butterworth, third order) was used to reduce data contamination due to power line noise. All epochs were visually inspected, and those contaminated by large artefacts due to head movement or muscle contractions were excluded from further analyses. The corresponding Force trials were also excluded. These trials constituted 2.0% of the total number of trials. Artefacts due to eye blinks or eye movements were subtracted using a validated method based on an Independent Component Analysis (Jung et al., 2000). In all datasets, independent components related to eye movements had a large EOG channel contribution and a frontal scalp distribution. As requested by one of the reviewers, we minimised the possible contribution of eye blinks and eye movements by re-referencing the EEG data to the average of all peripheral electrodes (i.e. Fp1, Fpz, Fp2, F7, F8, T3, T4, T5, T6, O1, Oz, O2). This peripheral average reference was preferred to the average reference across all electrodes because (1) we only recorded from 26 electrodes, and (2) these electrodes were unevenly distributed across the scalp, with a higher electrode density over the scalp vertex (i.e. where the largest part of the stimulus-evoked response is recorded). To match the sampling rate of the force timeseries, EEG epochs were finally downsampled to 1,000 Hz.

### 2.8 Time-frequency analysis of EEG and force timeseries

A time-frequency representation of each EEG and Force epoch was obtained using a windowed Fourier transform with a variable-width Hanning window moving in steps of 10 ms. The width of the Hanning window decreased linearly with frequency, from 500 ms (used to estimate the lowest frequency: 1 Hz) to 100 ms (used to estimate the highest frequency: 40 Hz). These widths are appropriate for identifying both high and low frequency responses evoked by salient stimuli while avoiding that estimates of post-stimulus responses are contaminated by pre-stimulus activity (Zhang et al., 2012; Hu et al., 2015).

This analysis yielded, for each single trial, power estimates *P(t,f)* at each *(t,f)* bin of the time-frequency plane extending from −1 to +3 ​s in the time domain, and from 1 to 40 ​Hz (in steps of 1 ​Hz) in the frequency domain. The magnitude of the stimulus-induced power changes [*PC(f,t)*] was estimated as follows:

PC(t,f) ​= ​[P(t,f) – R(f)] /[P(t,f) ​+ ​R(f)]

Where *P(t,f)* is the power spectral density at each time-frequency bin *(t,f)*, and *R(f)* is the average power spectral density of the signal enclosed within the prestimulus reference interval for each estimated frequency *f* (van Ede et al., 2010, 2011). The range of the prestimulus reference interval *t* was proportional to the frequency of interest, and was defined as:

-t1(f) < t(f) < -t2(f)

Where *t1* is twice the width of the time window used to estimate power at each frequency *f*, and *t2* is half of the width of the time window used to estimate power at each frequency *f*. The power values within this interval were averaged to obtain *R(f)*.

The time-frequency representation of induced change in oscillatory power computed as described above contains both phase-locked and non-phase-locked responses. To distinguish between phase-locked and non-phase-locked responses, we calculated the phase-locking value (PLV) (Lachaux et al., 1999), which represents a measure of phase consistency across trials, as follows:

$$PLV\left(t,f\right)=\left|\frac{1}{N}\overset{N}{\underset{r=1}{\sum }}e^{i\left(\emptyset_{t,f,r}\right)}\right|$$

where *N* is the number of trials, while ∅ is the phase calculated on each trial *r* at the time *t* and frequency *f*. Thus, the PLV is 1 if all trials are perfectly phase-locked at a given time-frequency bin. PLV were finally baseline corrected by subtracting the PLV of the prestimulus reference interval *R(f)* from each post-stimulus bin, to express stimulus-evoked *changes* in phase locking: the Phase Locking Change (PLC).

### 2.9 Statistical analysis

Two sets of statistical analyses were performed. The first treated EEG and Force independently, and aimed to identify significant stimulus-induced changes in the EEG and Force time-frequency spectra. The second set of analyses aimed to investigate the relationship between stimulus-induced changes in EEG and Force. Specifically, it tested whether trials associated with large EEG power were also associated with large Force power at the same latency and frequency (correlations “over trials”) (Cohen, 2014).

#### 2.9.1 Stimulus-induced EEG and force time-frequency modulations

For both EEG and Force data, single-trial time-frequency spectra belonging to the same experimental modality (i.e., somatosensory or auditory) were averaged together, thus yielding two *PC(t,f)* spectra for each participant.

To assess the consistency of stimulus-induced spectral modulations across participants, these averages were entered into a non-parametric cluster-based permutation testing (Maris and Oostenveld, 2007), which consisted of two steps. First, the values of each *PC(t,f)* bin were contrasted against zero (i.e. against baseline), using one-sample t tests. Next, bins significantly different from zero (*p* < 0.05) were clustered together if they were consecutive either in time or in frequency. When this analysis was conducted on the EEG data, clusters were also based on the spatial adjacency of the EEG channels. Specifically, a cluster had to be composed of at least two neighbouring *(t,f)* bins (either in time or in frequency) with a *p* value < 0.05 on at least three neighbouring EEG channels. When this analysis was conducted on the Force data, for which we had only one channel, only consecutivity in time domain or adjacency in the frequency domain were considered.

Once the clusters were identified, a significance test statistic of each cluster was computed by summing the t values of the bins composing it. Next, to assess the significance of each cluster, we used permutation testing against 0 (i.e. against baseline), to generate a random significance distribution (1000 permutations). This random distribution was used to define a threshold (*p* = 0.05) against which the actual significant clusters were assessed.

The phase locking change *PLC(t,f)* were analysed following the same procedure described above.

#### 2.9.2 Relationship between EEG and force time-frequency power

*Correlations over trials*. The relationship between EEG and Force *PC(t,f)* power spectra was analysed at the trial-by-trial level. With this analysis we tested whether the trial-by-trial variability in EEG power was related to the trial-by-trial variability of Force power. To achieve this, each EEG and Force time-frequency bin was smoothed over time using a sliding window of 10 time bins (corresponding to 100 ms). Next, for each time-frequency bin, we calculated a trial-by-trial correlation coefficient (Spearman's *r*_*s*_) between EEG and Force. This yielded, for each participant and EEG electrode, a time-frequency correlation matrix consisting of 240 × 40 (time x frequency) Spearman's *r*_*s*_ values (the total window time range was −0.4–2 s). Finally, these Spearman's *r*_*s*_ values were Fisher's z-transformed.

The across-participants consistency of *over trials* correlation matrices was assessed by using the same non parametric cluster-based permutation test discussed above (see *Stimulus-induced EEG and Force time-frequency modulations*), here contrasting the correlation coefficients vs. zero. Thus, resulting positive clusters would index significant positive correlations between EEG and Force power, while negative clusters would index negative correlations.

### 2.9.3 Test of lateralization (TOL)

When statistical analyses yielded significant clusters of stimulus-induced power changes (*PC*) or EEG-Force correlations (*r*_s_), we tested whether their scalp distribution was lateralised, using the following procedure. We first averaged the *PC* or *r*_*s*_ values separately for the left electrodes Cp3, C3 and FC3, and for the right electrodes Cp4, C4 and FC4, in each participant. The two resulting values were compared across participants using a paired-sample *t*-test. Negative t-values implied a lateralization towards the left hemisphere, while positive t-values implied a lateralization towards the right hemisphere. This test of lateralization is below referred to as TOL.

## 3 Results

**Fig. 2:**
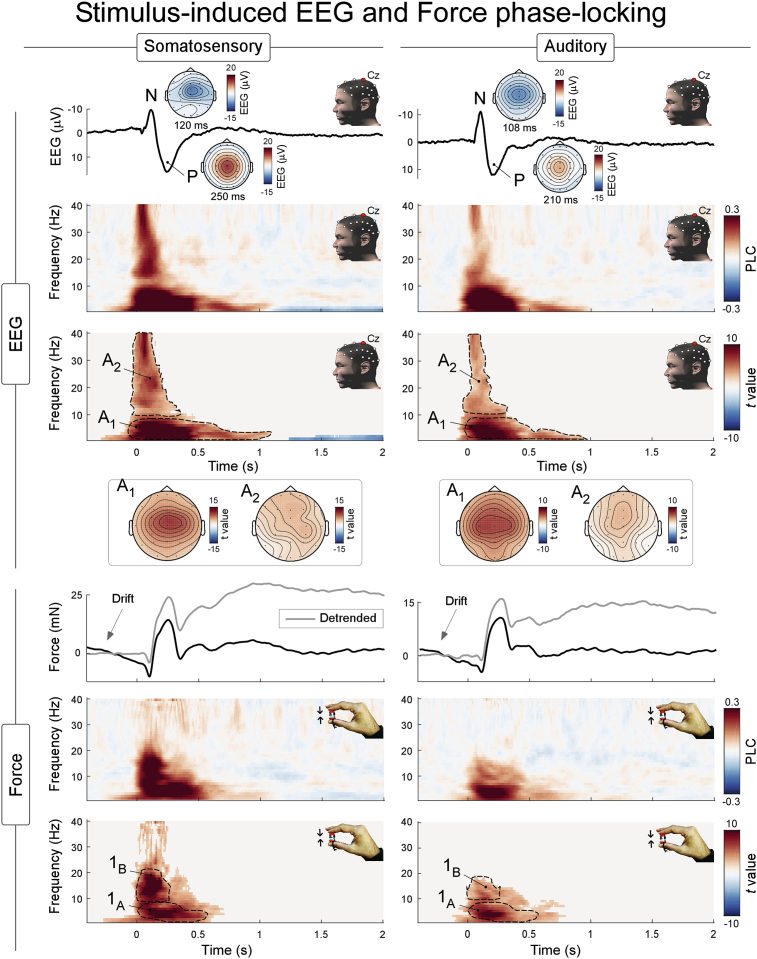
EEG (top) and Force (bottom) phase-locked modulations evoked by somatosensory (left) and auditory (right) stimuli. The waveforms represent the EEG amplitude (top) and Force magnitude (bottom) modulations in the time-domain. The bottom spectrograms represent the baseline-corrected phase locking value (i.e., the phase locked change; PLC) and the statistics assessing its consistency across participants (*t* value, resulting from a cluster-based permutation statistics), at electrode Cz. Scalp topographies of significant clusters are also provided. Dotted contours are for illustrative purposes; time-frequency intervals used to display topographies are detailed in the results section.

**Fig. 3:**
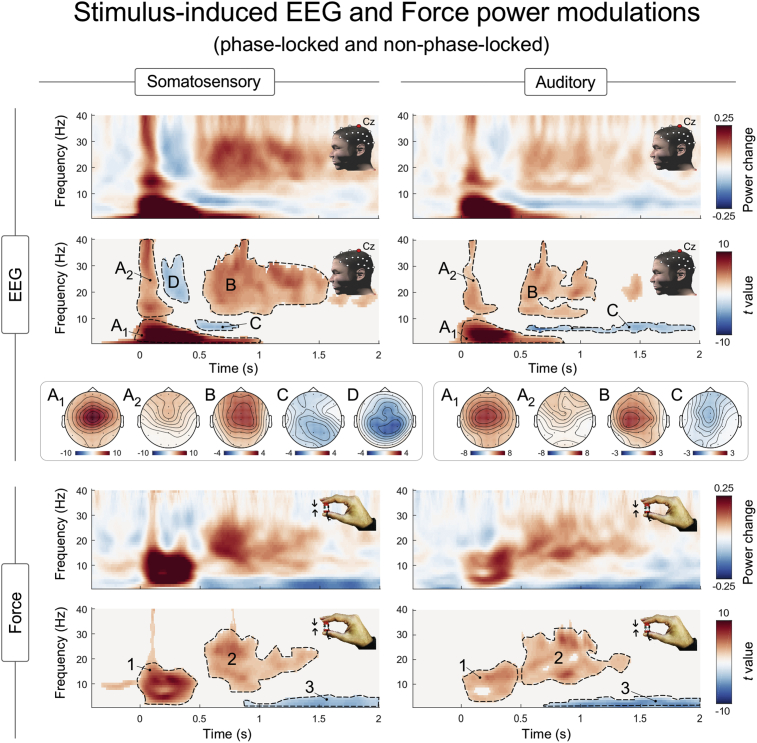
EEG (top) and Force (bottom) power modulations induced by somatosensory (left) and auditory (right) stimuli. The spectrograms represent the spectral power changes with respect to baseline and the statistics assessing the consistency across participants (*t* value, resulting from cluster-based permutation statistics) at electrode Cz in the time-frequency domain. Scalp topographies of significant clusters are also provided. Dotted contours are for illustrative purposes; time-frequency intervals used to display topographies are detailed in the results section.

Fig. 2 shows the results of the phase-locking change (PLC) analysis, which isolates phase-locked modulations of spectral power. Fig. 3 instead shows both phase-locked and non-phase-locked modulations of spectral power. Thus, modulations displayed in Fig. 3 but not in Fig. 2 are non-phase-locked. In the following sections, we mainly describe the results displayed in Fig. 3, which comprises all modulations. We sporadically refer to Fig. 2 when discussing phase-locked modulations.

### 3.1 Stimulus-induced modulations of EEG spectral power

The upper panels of Fig. 3 display the stimulus-induced modulations of EEG spectral power at electrode Cz, together with their cross-participants consistency and the corresponding scalp topographies (Fig. 3, upper panels). Both somatosensory and auditory stimuli elicited three significant modulations. These consisted of one phase-locked response, largely corresponding to the ERP observable in the time-domain (*ERP*, composed of two subclusters previously described as separate responses: A_1_: 0–0.9 s, 1–10 Hz, and A_2_: 0–0.2 s, 10–40 Hz; Mouraux et al., 2003; Iannetti et al., 2008), and two non-phase-locked responses: (i) an enhancement of beta-band oscillations (*β-enhancement*; cluster B: 0.5–1.5 s, ∼13–30 Hz), and (ii) a suppression of alpha-band oscillations (*α-suppression*; cluster C: ∼0.5–1.9 s, ∼8–10 Hz). In addition, only somatosensory stimuli induced an additional transient suppression of beta-band oscillations (*β-suppression*; cluster D: ∼0.2–0.3 s, ∼15–30 Hz).

The power increases A_1_ and A_2_ were modulations of low (1–10 Hz, 0–0.9 s) and high (10–40 Hz, 0–0.2 s) frequencies, respectively (Fig. 3, cluster-corrected significance: *p* < 0.001 [somatosensory stimulus], *p* < 0.001 [auditory stimulus]). The sub-cluster A_1_ is largely concomitant to the biphasic vertex potential observable in the time domain (Fig. 2, upper plots, PLC cluster-corrected significance: *p* < 0.001 [somatosensory stimulus], *p* < 0.001 [auditory stimulus]). Several previous reports have indeed demonstrated that this power increase is the time-frequency representation of the (transient) phase-locked biphasic vertex potential observed in the time domain (Mouraux et al., 2003; Hu et al., 2014). In support of this interpretation, we noticed (i) that the PLC analysis showed that these responses are clearly phase-locked (Fig. 2), and (ii) that the *ERP* time-frequency response and the vertex potential in the time domain have extremely similar scalp topographies (Fig. 2, upper plots). Instead, the second sub-cluster (A_2_) has a frontal and more widespread topography (Fig. 2). Therefore, A_2_ is a time-frequency response potentially reflecting a mixture of activities, likely entailing both early- and middle-latency phase-locked brain potentials (peaking up to 60 ms post-stimulus, for a review see (Cruccu et al., 2008; Pratt, 2012)) and minute non-phase-locked micro-saccades induced by the salient stimuli (the possible contribution of micro-saccades is suggested by the frontal distribution shown in Fig. 3).

Both somatosensory and auditory stimuli elicited a long-lasting *β-enhancement* (Fig. 3, cluster B; cluster-corrected significance: *p* < 0.001 [somatosensory], *p < *0.001 [auditory]), which was strongest over the central electrodes of the scalp, without a consistent lateralization across sensory modalities (TOL: *t* = 0.97 *p* = 0.33 [somatosensory], TOL: *t* = −1.40 *p* = 0.17 [auditory]).

Both somatosensory and auditory stimuli also elicited a *α-suppression* (Fig. 3, cluster C: cluster-corrected significance: *p = *0.057^1^ [somatosensory], *p* = 0.012 [auditory]). The scalp distribution of this *α-suppression* was different in the two modalities: following somatosensory stimuli it was slightly stronger on the central-parietal electrodes, while following auditory stimuli it was maximal on the midline, without clear evidence of lateralization (TOL: *t* = −0.72, *p* = 0.78 [somatosensory], TOL: *t* = 0.75 *p* = 0.45 [auditory]).

Only somatosensory stimuli induced an additional decrease of β power, i.e. a *β-suppression* (Fig. 3, cluster D; cluster-corrected significance: *p* = 0.024)*.* This *β-suppression* occurred between 0.2 and 0.3 s, just before the *β-enhancement*. Its scalp distribution was widespread over central-parietal electrodes and not lateralised (TOL: *t* = −1.12, *p* = 0.26).

### 3.2 Stimulus-induced modulation of force spectral power

The time-frequency analysis of Force timecourses revealed that both somatosensory and auditory stimuli elicited three significant modulations of power (Fig. 3, bottom panels). These consisted of a (i) phase-locked power increase at ∼0–0.5 s and 2–15 Hz (Fig. 3, bottom panel, cluster 1), (ii) a non-phase-locked power increase at ∼0.5–1.5 s and 13–30 Hz (Fig. 3, bottom panel, cluster 2), and (iii) a non-phase-locked power decrease at ∼0.75–2 s and 1–5 Hz (Fig. 3, bottom panel, cluster 3).

The first power increase (Fig. 3, cluster 1; 0–0.5 s and 2–15 Hz; cluster-corrected significance: *p* < 0.001 [somatosensory], *p* < 0.001 [auditory]) reflects the first two (transient) modulations composing the CMR observed in the time-domain and phase-locked to the stimulus onset (Novembre et al., 2018): the initial force decrease peaking at ∼100 ms post stimulus, and the following force increase peaking at ∼250 ms post stimulus (Fig. 2, bottom panels). This interpretation is confirmed by the PLC analysis, which showed that this power modulation was clearly phase-locked (Fig. 2, Fig. 3).

The power decrease (Fig. 3, cluster 3; ∼0.75–2 s and 1–5 Hz; cluster-corrected significance: *p* = 0.017 [somatosensory], *p* = 0.007 [auditory]) reflects the second (‘late’) increase of isometric force in the CMR detected in the time domain. This late force increase in the time domain is more visible after prestimulus detrending (Novembre et al., 2018), as shown in the gray waveform of Fig. 2. When the waveform is not detrended and the timecourse is transformed in the time-frequency domain, the late force increase appears as a decrease in power. This occurs because the baseline force signal is characterised by an ongoing negative “proprioceptive” drift (highlighted with an arrow in Fig. 2), reflecting the well-known decrease of exerted force throughout a holding task, a physiological phenomenon likely due to a gradual reduction of proprioceptive sensitivity (Wann and Ibrahim, 1992; Wolpert et al., 1998; Desmurget et al., 2000; Nazir et al., 2017). Given that the ongoing proprioceptive drift is reduced by the stimulus, after baseline correction a decrease of power at what is likely the frequency of the prestimulus drift appears.

Interestingly, the time-frequency analysis of the force timecourse also disclosed a modulation that did not simply reflect the time-frequency counterpart of the CMR observed in the time domain. Indeed, both somatosensory and auditory stimuli induced a second power increase (Fig. 3, cluster 2; 0.5–1.5 s and 13–30 Hz; cluster-corrected significance: *p* < 0.001 [somatosensory], *p* < 0.001 [auditory]). Since this modulation was not phase-locked, as shown by the PLC analysis (Fig. 2), it was not visible after across-trial averaging in the time domain. This modulation of power had a time-frequency distribution remarkably similar to the *β-enhancement* observed in the EEG data (cluster ‘B’ in the upper panels of Fig. 3), raising the intriguing possibility that the two phenomena are related to one another. This possibility was explored in the analysis of the relationship between the Force and the EEG power spectra.

### 3.3 Relationship between EEG and force modulations

*Over-trials correlations.* This analysis explored the correlation between the trial-by-trial variability of EEG and Force power spectra: it explored whether trials associated with large EEG power also had large Force power at the same latency and frequency. There were two significant clusters showing positive trial-by-trial correlations.

**Fig. 4:**
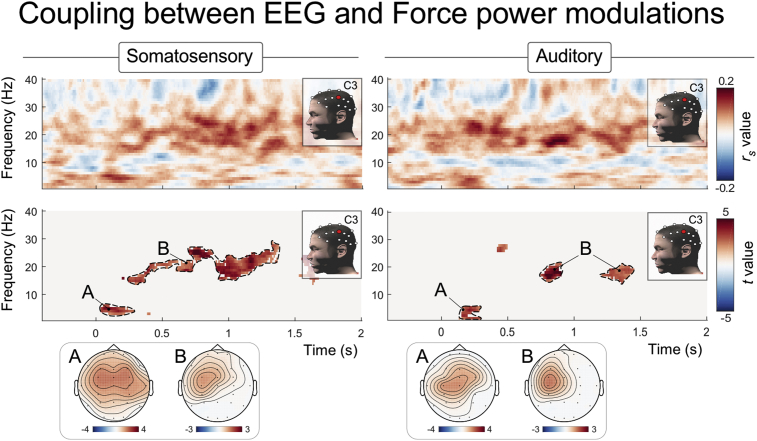
Coupling between EEG and Force spectral modulations. The spectrograms represent the correlation strength between the two spectra (fisher-transformed Spearman's *r*_*s*_ value) and the statistics assessing the consistency across participants (*t* value, resulting from a cluster-based permutation statistics) at electrode C3 in the time-frequency domain. Scalp topographies of significant clusters are also provided. Spectrograms represent the results from a correlation over trials, assessing whether trials associated with large EEG power were also associated with large Force power at the same latency and frequency. Dashed contour lines are for illustrative purposes; time-frequency intervals used to display topographies are detailed in the results section.

The first cluster (∼0–0.3 s and 3–5 Hz; cluster A, Fig. 4, lower panels; cluster-corrected significance: *p* < 0.001 [somatosensory], *p* = 0.02 [auditory]) reflected the previously described correlation between the negative and positive VW in the time domain, and the first force increase of the CMR (Novembre et al., 2018). These correlations are reminiscent of the lateralised distributions of the correlation between the positive VW and the force increases that we previously observed in the time domain, and are further discussed below (topographies A-D in Fig. 5 of Novembre et al., 2018) (TOL: *t* = 0.21, *p* = 0.83 [somatosensory], *t* = −2.07, *p* = 0.047 [auditory]).

The second cluster (∼0.3–1.5 s [somatosensory], ∼0.75–1.5 s [auditory], and 15–28 Hz; cluster B, Fig. 4, lower panels; cluster-corrected significance: *p* < 0.001 [somatosensory], *p* = 0.008 [auditory]) reflected the trial-by-trial correlation between the stimulus-induced non-phase-locked *β-enhancement* in the EEG (Fig. 3, cluster B, top panel) and the concurrent and isofrequent power increase in the force (Fig. 3, cluster 2, bottom panel). Thus, trials with large *β-enhancement* also had large power in force oscillations at the same latency and frequency. In both the auditory and somatosensory modality, this second cluster of correlations was clearly lateralised, and strongest on the electrodes overlying the hemisphere contralateral to the hand exerting the force (topographies B in bottom panels of Fig. 4) (TOL: *t* = −3.30, *p* = 0.002 [somatosensory], *t* = −3.65, *p* = 0.001 [auditory]).

## 4 Discussion

We characterised the modulations of electrocortical (EEG) and muscular (Force) spectral power induced by salient environmental stimuli, as well as the relationship across trials between such modulations. We report two main results. First, somatosensory and auditory stimuli elicited similar modulations of spectral power in both EEG and Force measures. In each measure, modulations were both phase-locked and non-phase-locked. Second, we identified two time-frequency regions where stimulus-induced EEG and Force modulations were coupled. One region corresponded to the phase-locked modulations that have been already demonstrated to be coupled in the time domain (Novembre et al., 2018). The second region corresponded to the non-phase-locked increase of power observed both in the EEG and in the Force at approximately 20 Hz, and ~0.5–1.5 s post-stimulus. These results show that sudden environmental stimuli lead to a strong coupling between cortical activity and exerted force not only in phase-locked responses but also in β-band, non-phase-locked cortical oscillations. Thus, stimulus-induced corticomuscular coupling occurs across at least two different types of cortical activities.

### 4.1 Supramodal modulation of EEG spectral power

Somatosensory and auditory stimuli elicited similar patterns of EEG activity. These patterns consisted of both phase-locked and non-phase-locked (Fig. 2, Fig. 3) modulations of spectral power.

*Phase-locked ERP.* The stimulus-induced phase-locked response (*ERP*, sub-cluster A_1_) entailed a short latency (0–0.9 s) power increase of low (1–10 Hz) frequencies. This phase-locked modulation is the time-frequency representation of the biphasic EEG vertex potentials detected at the same latency in the time domain (Davis, 1939; Bancaud et al., 1953; Carmon et al., 1976; Naatanen and Picton, 1987). Several previous reports have indeed demonstrated that the vertex potential can be elicited by sudden stimuli of distinct modalities (Mouraux and Iannetti, 2009; Liang et al., 2010; Valentini et al., 2011), and that such potential has a time-frequency representation similar to the one we observed here (Mouraux et al., 2003; Mouraux and Iannetti, 2008; Hu et al., 2014, 2015; Hu and Iannetti, 2019). In support of this view, the scalp distribution of the *ERP* cluster was centrally distributed and strongest over the vertex (Fig. 2, Fig. 3), like the vertex potential in the time domain (Fig. 2).

*Non-phase-locked α-suppression and β-suppression.* The stimuli also elicited non-phase-locked decreases of spectral power: an *α-suppression* induced by both the somatosensory and the auditory stimuli, and a *β-suppression* only induced by somatosensory stimuli (Fig. 3). These non-phase-locked suppressions have been repeatedly reported. Both *α*- and *β-suppressions* are elicited by somatosensory stimuli, with a maximum above the primary sensorimotor cortex^2^ (Salenius et al., 1997a,b; Cheyne et al., 2003; Bauer et al., 2006; van Ede et al., 2011). In contrast, auditory stimuli have been shown to elicit only *α-suppression*, which is commonly described as having a central distribution consequent to activity localized in the bilateral auditory cortex (like in the current results, Fig. 3) (Lehtela et al., 1997; Weisz et al., 2011; Hartmann et al., 2012; Obleser and Weisz, 2012). From a functional perspective, both *α*- and *β-suppressions* have been associated with perceptual and attentional processes, with stronger suppression associated with better auditory and somatosensory discrimination (Foxe and Snyder, 2011; van Ede et al., 2011; Obleser and Weisz, 2012).

*Non-phase-locked β-enhancement.* The most interesting time-frequency EEG response was the non-phase-locked *β-enhancement*, which was elicited by both somatosensory and auditory stimuli (Fig. 3). This *β-enhancement* is commonly observed following somatosensory stimuli, and, in this modality, it is often described as a “rebound” because it typically follows a preceding *β-suppression* (described in the previous paragraph) (Salmelin and Hari, 1994; Cheyne et al., 2003; Bauer et al., 2006). Its scalp distribution is central, and usually stronger over the hemisphere contralateral to the stimulated limb (Neuper and Pfurtscheller, 2001; Pfurtscheller et al., 2001; Neuper et al., 2006). The *β-enhancement* is usually not observed in response to auditory stimuli (but see (Leventhal et al., 2012)), unless participants have to perform a task entailing an association between the auditory stimulus and movements or actions (Caetano et al., 2007; Fujioka et al., 2012). This is not surprising, given that β oscillations have been repeatedly shown to be associated with motor functions (Pogosyan et al., 2009; Novembre et al., 2017). For instance, β oscillations originating from somatosensory cortices typically decrease during movement, and rebound following movement termination (Pfurtscheller and Lopes Da Silva, 1999). More importantly with respect to our current results, β oscillations increase while holding a posture (Gilbertson et al., 2005), and, as further discussed in the next paragraph, can even be recorded directly from the muscles involved in the motor task using EMG (Baker et al., 1997; Kilner et al., 1999). Thus, considering that our participants had to exert an isometric force (Fig. 1), both the somatosensory and the auditory-induced *β-enhancement* that we observed likely reflect the impact of salient environmental stimuli on the ongoing dynamics of the motor system.

### 4.2 Supramodal modulation of force spectral power

Somatosensory and auditory stimuli elicited virtually identical spectral modulations of Force. They consisted of one phase-locked and two non-phase-locked responses (Fig. 2, Fig. 3).

The previously-described first two CMR components in the time domain (Novembre et al., 2018) fully explain the current observation of a phase-locked modulation of spectral power occurring in the 500 ms following the stimulus, and divided in two subclusters: one with a time-frequency maximum at 0.1 s and ∼15 Hz, reflecting the first force decrease in the time domain (subcluster ‘1_B_’ in Fig. 2), and one with a time-frequency maximum at 0.25 s and ∼4 Hz, reflecting the following force increase in the time domain (subcluster ‘1_A_’ in Fig. 2). In contrast, the last more sustained CMR component explains the low-frequency non-phase-locked modulation of spectral power observed from ∼0.75 s following the stimulus (cluster ‘3’ in Fig. 3).

More remarkably, the time-frequency analysis revealed an additional non-phase-locked increase of spectral power in a time-frequency region similar to the one containing the cortical *β-enhancement* (clusters ‘B’ and ‘2’ in Fig. 3). This spectral modulation, occurring at 0.5–1.5 s with a mean frequency of ∼20 Hz, was elicited by both auditory and somatosensory stimuli. Importantly, this modulation cannot be directly explained by any of the CMR components observed in the time domain as it was non-phase-locked, and therefore not detectable by the phase locking change (PLC, Fig. 2). Both increases and decreases of oscillatory activity in frequencies overlapping with the cortical β (13–30 Hz) have been identified in the EMG during a number of sensorimotor tasks (Baker et al., 1997; Kilner et al., 1999; van Ede and Maris, 2013). Such EMG activity has been shown to result in an actual finger “microtremor” at the same frequency, which can be detected by isometric force transducers (McAuley et al., 1997; Gilbertson et al., 2005; Airaksinen et al., 2015). It must be noted that these earlier observations of oscillatory activity of both EMG and force at ∼20 Hz were made in studies entailing purely motor tasks. Here not only we confirm the existence of such 20 Hz activities, but we also show that they are enhanced by environmental salient stimuli. To better understand the functional significance of these oscillations, we need to consider their relationship with the concomitant stimulus-induced cortical activity.

### 4.3 Coupling of EEG and force spectral modulations

To explore the relationship between EEG and Force spectral modulations, we correlated the power of the two measures over trials. This analysis tested whether trials with large modulations of cortical oscillatory power also entailed large modulations of Force oscillatory power at the same latency and frequency. These results indicated that, following both somatosensory and auditory stimuli, EEG and Force signals became coupled in two separate regions of the power spectra.

The first coupled time-frequency region was maximal at ∼4 Hz and ∼0.25 s (Fig. 4). This result is the time-frequency representation of our previous observation that EEG vertex potentials and Force are coupled in the time domain. More precisely, the coupling we observed in the time-frequency domain reflects the trial-by-trial correlation between the amplitude of the P vertex potential and the first force increase elicited by somatosensory stimuli (Fig. 5 in Novembre et al., 2018). The current results additionally show that the same trial-by-trial EEG-Force relationship also holds when the cortical and CMR responses are elicited by auditory stimuli (Fig. 4). The topography of this correlation was centrally distributed over the electrodes covering sensorimotor regions, and, following the auditory stimuli, was stronger in the hemisphere contralateral to the hand exerting the force task. Although the time-frequency EEG response at ∼4 Hz lumps together the N and the P waves of the vertex potential (whose correlations with the force signal in the time domain have meaningfully different scalp distribution, see Fig. 5 in Novembre et al., 2018), these time-frequency observations are consistent with an effect of salient sensory stimuli on a number of motor behaviours (Fautrelle et al., 2010; Perfiliev et al., 2010; Pruszynski et al., 2010, 2016; Pruszynski, 2014; Wood et al., 2015; Moayedi et al., 2015; Gu et al. 2016, 2017; Scott, 2016; Kilintari et al., 2018; Reuter, 2018). These results are also consistent with the notion that the vertex potential is elicited by a network of cortical regions comprising the anterior cingulate (Mouraux and Iannetti, 2009, 2018), which is known to be associated with motor control (Shackman et al., 2011; Caruana et al., 2018) via direct projections to the primary motor cortex and even spinal motor neurons (Dum and Strick, 1991, 2002).

The second time-frequency region showing a coupling between EEG and Force was maximal at ∼20 Hz, and occurred in a time window ranging between 0.5 and 1.5 s post-stimulus (Fig. 4, cluster B). This is the most important result of the current study, as it did not stem from phase-locked responses, and therefore could not have been observed in the time-domain. This observation establishes a direct link between the cortical *β-enhancement* and the concomitant increase of Force spectral power at the same frequency. The topography of this coupling was revealing, as it was maximal over the electrodes overlying the sensorimotor regions *contralateral* to the hand performing the force exertion task (Fig. 4, cluster B). Thus, these results expand what we previously observed in the time domain, and demonstrate that the stimulus-induced corticomuscular coupling is driven by two fundamentally different features of the cortical response elicited by the salient stimulus.

A coupling between *β* cortical oscillations and isofrequent oscillations in both the EMG and the force signal has previously been observed during voluntary movements and constant isometric tasks (Conway et al., 1995; Baker et al., 1997; McAuley et al., 1997; Salenius et al., 1997a,b; Mima and Hallett, 1999; Gross et al., 2000; Airaksinen et al., 2015; Bourguignon et al., 2017; Ushiyama et al., 2017). An influential account of these observations is that cortical β oscillations promote the postural “status quo”, i.e. the maintenance of a steady motor output (Gilbertson et al., 2005; Androulidakis et al., 2006; Engel and Fries, 2010). In the context of our task, where such oscillations were elicited by sudden sensory stimuli, this account could suggest that the observed EEG-Force coupling in the β-band might reflect the nervous system's attempt to achieve motor stability following the force perturbation reflected by the first two components of the CMR, on the basis of a transient change in proprioceptive afference. From this perspective, the β-band coupling would be caused by, and therefore be part of, the CMR itself. In line with this reasoning, another study showed that auditory and visual distractors transiently modulate isometric force,^3^ and that this modulation is followed by a longer-lasting increase of MEG-EMG *β*-band coupling, which was interpreted as a recalibration of the ongoing (tonic) corticospinal coupling (Piitulainen et al., 2015).

Alternatively, it is possible that the β-band coupling is caused directly by the stimuli, independently of the earlier components of the CMR. This interpretation is in agreement with the observation of a clear *β-enhancement* in motor tasks that rely on sensory stimulation, such as the maintenance or revision of motor plans on the basis of sensory information (Tan et al., 2016), or the sudden interruptions of on-going behaviour in response to unexpected or infrequent events (Swann et al., 2009; Ray et al., 2012; Fischer et al., 2016; Wessel et al., 2016; Wagner et al., 2018; Muralidharan et al., 2019).

Obviously, future research is needed to understand better the functional significance of these corticomuscular couplings. This should be done bearing in mind that these couplings, traditionally observed during purely motor tasks (Conway et al., 1995; Baker et al., 1997; Salenius et al., 1997a,b; McAuley et al., 1997; Mima and Hallett, 1999; Gross et al., 2000, 2002; Kristeva-Feige et al., 2002; Jerbi et al., 2007; Airaksinen et al., 2015; Ushiyama et al., 2017; Bourguignon et al., 2017), can in fact be enhanced by salient sensory stimuli (Piitulainen et al., 2015; Novembre et al., 2018). Thus, to understand these neurophysiological phenomena, it is important to consider the functional continuum between sensory and motor systems.

## Conflicts of interest

The authors declare no conflict of interest.

## Footnotes

1 We report this result as the cluster-based permutation test used to detect it is very conservative. Moreover, we highlight that this response has been observed in other studies, as detailed in the Discussion.

2 Note that this should not be taken as direct evidence that this signal is generated in the primary somatosensory cortex.

3 We note that Piitulainen et al. (2015) interpreted these transient force modulations as “covert startle-like” responses, something that we recently indicated not to be the case (Novembre et al., 2018).
